# Editorial to “The Japanese catheter ablation registry (J‐AB): Annual report in 2022”

**DOI:** 10.1002/joa3.13176

**Published:** 2024-11-04

**Authors:** Germaine Loo, Chi Keong Ching

**Affiliations:** ^1^ Department of Cardiology National Heart Centre Singapore Singapore Singapore

In recent years, several nationwide registries for patients undergoing catheter ablation have been published.^1^ This provides perspective on real‐world practice, reporting in depth information on the current trends, efficacy, and safety data on catheter ablation. There is a rise in prevalence of atrial fibrillation and other arrhythmias, due to global ageing populations, increased patient comorbidities, and technological advancements in arrhythmia detection.^2^ This translates to an increasing volume and trend of patients undergoing catheter ablation—most prominent for atrial fibrillation. Comparing data from the Japanese Catheter Ablation Registry (J‐AB) from 2018 to 2022, there has been an increasing overall number and proportion of atrial fibrillation ablation cases: 40, 422 cases (72.8%) versus 68, 378 cases (75.9%), respectively.^1^

The biggest strength of the J‐AB registry lies in its numbers, recording the acute procedural characteristics and outcomes of a total of 90,042 cases, which is one of the largest reported nationwide registries to date. Knowing the ablation trends and volume would aid healthcare cost projection at both hospital and nationwide levels. In addition, this would serve as an important data repository for investigators to explore further regarding the mechanisms and treatment of arrhythmias.

Marked advancements in the field of catheter ablation over the past decades have led to improvements in durability and safety of catheter ablation.^3^ The development and utilization of electro‐anatomical mapping systems, novel catheters and energy sources for ablation, as well as innovative ablation strategies have revamped the catheter ablation landscape. Cryoablation ablation has showed high acute procedural success and lower incidence of procedural complications compared to radiofrequency ablation (RFA).^3^ Despite this, looking at the current data from the J‐AB 2022 registry, majority of pulmonary vein isolation (PVI) ablation were performed with RFA alone (72.7%), with the rest utilizing alternative techniques with or without concurrent RFA.

Pulsed field ablation (PFA), utilizing electric pulses to produce non‐thermal irreversible electroporation and cell death, is emerging as a suitable alternative to achieve PVI. The penta‐spline catheter design allows for rapid and efficient PVI, and the integration of PFA catheters with current 3‐D electro‐anatomical mapping systems can reduce fluoroscopy duration. Although prospective trials have validated the clinical efficacy of PFA, large randomized controlled trials comparing radiofrequency ablation to PFA are awaited.^4^ We hypothesize that there will be a greater trend towards using PFA for PVI in the coming years and in turn increase the volume of AF ablation.

Results from the J‐AB registry showed an acute procedural success rate of 99.6% for first time PVI for atrial fibrillation and 99.3% for cavo‐tricuspid isthmus dependent atrial flutter. Acute overall complication rates from catheter ablation were low at 2.3%, with the most common being major bleeding complications at 0.9%. Mortality rate related to ablation therapy was less than 0.1%. This result is similar to that reported in other nationwide registries.^1^ The reported rates serve as a benchmark for newer centers to assess their quality of catheter ablation. Perhaps what can be improved upon is the collection of procedural duration and fluoroscopic duration to further aid the evaluation of procedural safety for both patients and operators.

Finally, there are still many unanswered questions pertaining to catheter ablation. Additional information, such as ablation lesion sets beyond PVI in atrial fibrillation, ablation energy sources, utilitization of electroanatomical mapping system and intraprocedural imaging statistics will further enrich this registry. Similarly, long term outcome data regarding arrhythmia burden post ablation, usage of antiarrhythmic drugs, and anticoagulation therapy will add to its strength. These data may help bridge knowledge gaps in catheter ablation for management of arrythmias. Nevertheless, the J‐AB registry serves a key role in providing a contemporary understanding in the trends and outcomes of Asian patients undergoing catheter ablation.

## CONFLICT OF INTEREST STATEMENT

There are no conflict of interest for all authors.
